# A cross-cultural comparison of educational video quality on college students’ anxiety and depression: a cross-sectional content analysis of YouTube and Bilibili

**DOI:** 10.3389/fpubh.2026.1783552

**Published:** 2026-03-11

**Authors:** Jianbo Xu, Qiaoli He, Jing Chen, Xianrong Peng, Jiachen Liang

**Affiliations:** 1 School of Foreign Studies, Xiangnan University, Chenzhou, Hunan, China; 2 College of Medical Imaging Laboratory and Rehabilitation, Xiangnan University, Chenzhou, Hunan, China; 3 Academic Affairs Office, Xiangnan University, Chenzhou, Hunan, China; 4 Shaanxi University of Chinese Medicine, Xianyang, Shaanxi, China

**Keywords:** anxiety, Bilibili, cross-cultural, depression, online platforms, quality assessment, university students, YouTube

## Abstract

**Objective:**

To systematically compare the quality of educational videos about anxiety and depression among university students on YouTube and Bilibili, and to provide evidence-based guidance for cross-cultural digital mental-health education.

**Methods:**

Before 20 November 2025, we searched YouTube and Bilibili with English and Chinese keywords and collected the first 100 videos returned by default ranking on each platform. After applying inclusion and exclusion criteria, the remaining videos were evaluated by a third assessor in a double-blind manner using the Video Information and Quality Index (VIQI), the Global Quality Score(GQS)and the modified DISCERN (mDISCERN) scales to assess scientific accuracy, safety and educational value. Platform differences were analyzed with non-parametric tests and correlation analyses.

**Results:**

The final sample comprised 80 YouTube and 77 Bilibili videos. Median views, likes, and comments were markedly higher on Bilibili (*p <* 0.05). Verified accounts supplied 43.75% of YouTube content but only 28.57% of Bilibili content; licensed mental-health professionals appeared in fewer than 6% of videos on either platform. YouTube favoured television-style or documentary formats, whereas Bilibili relied heavily on single-speaker narratives and animations. YouTube outperformed Bilibili on overall VIQI, GQS, and mDISCERN scores (*p <* 0.01). On Bilibili, high user engagement correlated moderately to strongly with quality, yet absolute quality scores remained low.

**Conclusion:**

Platform architecture, not popularity, drives content quality. YouTube’s longer, institution-produced videos set the benchmark, whereas Bilibili trades scientific rigor for real-time chat and high engagement. Both sites remain short of licensed professionals. To prevent digital platforms from amplifying student anxiety, we recommend (a) embedding a quality-weighted algorithmic boost and (b) a sustained “verified expert + student co-creation” pipeline that disseminates evidence-based content at scale.

## Introduction

The undergraduate years mark the bridge between adolescence and adult life (1). During this interval, rapid changes in academic demands, social networks, and career prospects can exert lasting effects on emotional well-being (2). Freshmen and sophomores typically struggle to rebuild friendships and manage coursework, whereas juniors and seniors confront job-market pressure, repeated rejection, and uncertainty about the future (1). The cumulative burden of academic overload, social adjustment, and vocational ambiguity has pushed depression and anxiety rates steadily upward, turning student mental health into a global public-health priority (3, 4).

In the digital era, most people turn to online video for health information (5). The internet dissolves the traditional divide between clinician and patient, turning users from passive recipients into active seekers and giving them a larger role in managing their own health. Yet the same openness creates risk: variable quality and widespread misinformation can distort decisions and undermine care. Raising the scientific and educational standard of online health videos is therefore essential to help the public form accurate beliefs and make sound choices.

YouTube, the world’s largest video-sharing site (6), and Bilibili, the leading platform in China (7), have both become key channels for mental-health education. Algorithm design, cultural context, and audience expectations differ between the two services, so videos on the same topic can vary markedly in depth, style, and reliability. To date, no study has systematically compared how the two platforms present educational content on university students’ anxiety and depression.

This study uses YouTube and Bilibili as natural laboratories. Applying multi-dimensional quality tools, we systematically examine educational videos on university anxiety and depression for scientific accuracy, safety, and instructional value. Our goals are three-fold: (1) map cultural differences in how Western and Chinese platforms present mental-health content; (2) gauge video quality and its potential impact on students; and (3) supply evidence-based guidance for users, educators, and policymakers to standardize health communication on social media.

## Materials and methods

### Ethical considerations

All data were extracted from publicly available videos on YouTube and Bilibili. The study did not collect identifiable personal information, clinical records, human tissue, or animal data, so ethics approval was not required (8, 9).

### Video collection

On 20 November 2025 we searched YouTube with the phrase “psychological anxiety and depression among college students” and searched Bilibili with the Chinese equivalent “大学生心理焦虑抑郁.” The Chinese wording is widely used in both academic and popular contexts and is identical in simplified and traditional characters. All accounts were logged out and browsing histories cleared to avoid personalized recommendations. Videos were retrieved by default ranking with no additional filters. Clips published within the previous 7 days were excluded because their view and like counts are unstable; promotional videos were also removed. The first 100 videos from each platform were retained, as previous work shows that content beyond this point has negligible influence on the results.

### Video characteristics

On 20 November 2025 we extracted each video’s metadata: duration, views, likes, comments, bookmarks, and shares. YouTube does not disclose bookmark or share counts; these fields were recorded as missing for that platform.

### Uploader characteristics

That same day we recorded the unique identifier code of each uploader, the number of followers, and the status of their account verification.

### Style assessment

Recording style was classified as monologue, Q&A, slide/lecture, animation/motion graphic, clinical setting, TV/documentary, or other.

### Quality assessment

From 20 to 27 November 2025 two reviewers independently rated each retained video; disagreements were resolved by a senior third adjudicator. Quality was assessed with the Video Information and Quality Index (VIQI), the Global Quality Score (GQS), and the modified DISCERN (mDISCERN).

The VIQI (10) evaluates four domains: (1) flow of information, (2) accuracy, (3) production quality (images, animations, interviews, captions, and summary, 1 point each, maximum 5), and (4) precision (title–content match). Each item is scored 1–5, with higher values indicating better quality.

The GQS (11) is a single-item, five-point scale that rates overall video quality from 1 (poor) to 5 (excellent).

mDISCERN (12), adapted from the DISCERN instrument, is designed for video appraisal (13). It poses five yes–no questions about reliability; each “yes” earns 1 point. Higher totals indicate greater reliability.

### Statistical analysis

All analyses were run in IBM SPSS 24.0. Normally distributed continuous variables are reported as mean ± SD; non-normal variables are presented as median (Q1, Q3) with range. Group comparisons for non-normal data used the Mann–Whitney U test. Categorical variables are summarized as counts and percentages. Spearman’s rho (r) assessed the association between audience engagement and video quality. Correlation strength was interpreted as negligible (|r| ≤ 0.2), weak (0.2 < |r| ≤ 0.4), moderate (0.4 < |r| ≤ 0.6), strong (0.6 < |r| ≤ 0.8), or very strong (|r| > 0.8). Two-tailed *p <* 0.05 was considered statistically significant.

## Results

### Video characteristics

**Figure 1:**
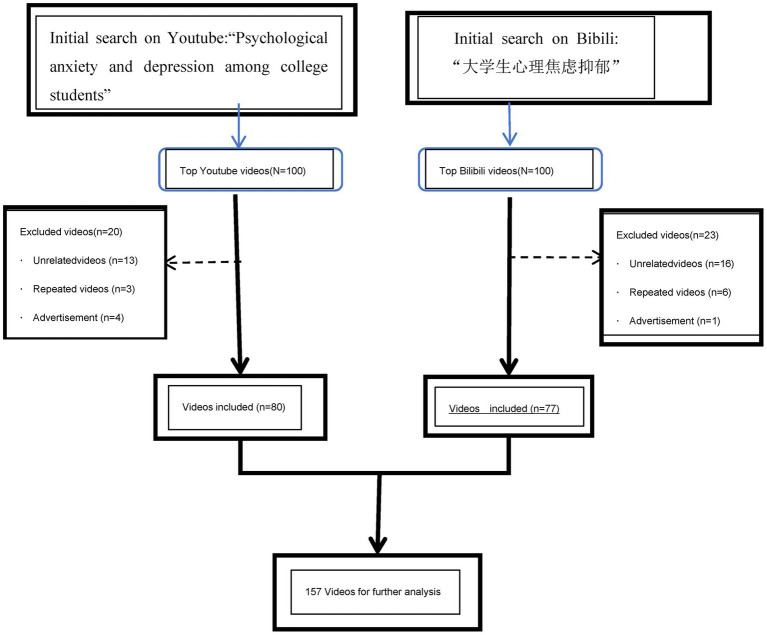
Video collection strategy and video filtering program.

After deduplication and relevance screening, 80 YouTube videos and 77 Bilibili videos were retained for analysis (Figure 1). All YouTube clips were in English or carried English subtitles; all Bilibili clips were in Chinese or carried Chinese subtitles. Table 1 summarizes their features. Because YouTube lacks “favorite-coin-share” metrics, those variables could not be compared. Among the comparable indices, views, likes, and comments differed significantly between platforms (*ρ* < 0.42, *p <* 0.05), whereas duration did not (*p* > 0.05). In absolute terms, Bilibili exceeded YouTube in median views, likes, and comments (*p <* 0.05); YouTube showed the lowest overall engagement.

**Table 1:** Basic characteristics of the video [M (Q1, Q3)].

Platform characteristic	YouTube (*N =* 81) M (Q1, Q3)	Bilibili (*N =* 77) M (Q1, Q3)	Z	*P*
Video length(s)	443.5 (232.75, 1022.50)	501 (214.5, 711)	−1.103	0.270
Views	1077.50 (66.50, 74697.25)	25,000 (2,150, 117,500)	−3.060	0.002
Thumbs up	8.5 (0, 1220.50)	755 (60, 4217.5)	−4.244	0.000
Comments	0 (0, 76.25)	95 (10.5, 349)	−4.397	0.000
Collections	NA	1,101 (58.5, 5,796)		
Shares	NA	161 (32, 2043)		
Coin	NA	131(18.5, 1216.5)		

Q1: First quartile; Q3: third quartile; NA: Not Available; Z: The z-value is obtained from the Wilcoxon signed-rank test, which is employed for non-parametric comparisons. P, *p* < 0.05 were considered statistically significant.

### Uploader characteristics

We identified 68 unique uploaders on YouTube and 74 on Bilibili (Table 2). Collectively, YouTube uploaders commanded 412,974,410 followers versus 43,429,140 for their Bilibili counterparts. Verified accounts supplied 35 (43.75%) YouTube videos and 22 (28.57%) Bilibili videos. Among the verified, licensed psychologists were scarce (five on YouTube, three on Bilibili); most verifications were held by individual creators (44 on YouTube, 55 on Bilibili), while not-for-profit institutions (hospitals, departments, or societies) accounted for only a handful. The remaining uploaders—51.6% on YouTube and 71.4% on Bilibili—lacked any platform verification.

**Table 2:** Uploader characteristics.

Platform characteristic	YouTube (*N =* 81)	Bilibili (*N =* 77)
Number of uploaders	68	74
Number of certifications	35 (43.75%)	22 (28.57%)
Certified practicing physician	5	3
Certified individual	28	13
Unverified	44	55

Table 3 shows the distribution of video styles. The two platforms display distinct genre profiles: YouTube is dominated by television-style or documentary formats (*N =* 41), followed by single-speaker narratives (*N =* 18) and slide-based lectures (*N =* 13). In contrast, Bilibili favors single-speaker monologues—often lengthy (*N =* 25)—with animated or motion-graphic pieces ranking second (*N =* 23); only two videos used a question-and-answer format.

**Table 3:** Video style.

Platform characteristic	YouTube (*N =* 81)	Bilibili (*N =* 77)
TV show/documentary	41	8
Solo narration	18	25
PPT/class	13	7
Animation/action	7	23
Questions and answers (Q&A)	0	2
Others	1	12

### Video quality

Overall, YouTube delivered the highest-quality content, outperforming all other platforms in VIQI total score, GQS, and mDISCERN total score (Table 4, *p <* 0.05).

**Table 4:** Quality assessment of videos on YouTube and Bilibili related to college students’ psychological anxiety/depression [Mean ± SD/M(Q1, Q3)].

Platform scores	YouTube (*N =* 81) M(Q1, Q3)	Bilibili (*N =* 78) M(Q1, Q3)	t/Z	*p*-value
mDISCERN-1	1 (1, 1)	1 (1, 1)	−0.027	0.978
mDISCERN-2	1 (1, 1)	1 (1, 1)	−1.428	0.153
mDISCERN-3	1 (1, 1)	1 (0, 1)	−4.907	0.000
mDISCERN-4	1 (0, 1)	1 (0, 1)	−2.331	0.020
mDISCERN-5	1 (0, 1)	0 (0, 0.5)	−4.310	0.000
mDISCERN-sum	4 (3.25, 5)	4 (2, 4)	−4.497	0.000
*GQS*	4 (3, 5)	3 (3, 4)	−3.688	0.000
*VIQI-1*	2 (1, 4)	3 (2, 4)	−2.861	0.004
*VIQI-2*	4 (3, 4)	3 (2, 4)	−3.549	0.000
*VIQI-3*	3(3, 4)	3 (2, 3)	−4.498	0.000
*VIQI-4*	4 (3, 5)	3 (2, 4)	−4.813	0.000
*VIQI-sum*	13.33 ± 3.714	11.75 ± 3.487	−2.731	0.007

SD, Standard Deviation; Q1, First quartile; Q3, third quartile; t, The *t*-value is derived from an independent samples *t*-test, which is utilized to compare the means between groups. Z: The *z*-value is obtained from the Wilcoxon signed-rank test, which is employed for non-parametric comparisons. P, *P* < 0.05 were considered statistically significant.

### Correlation analysis

On YouTube, VIQI scores correlated significantly with views (moderate), follower count (weak), likes (moderate), comments (weak), and video length (moderate; all *p <* 0.05). GQS and mDISCERN scores, in contrast, were significantly associated only with longer videos (moderate; *p <* 0.05) and showed no relationship with any other engagement metric.

On Bilibili, VIQI correlated significantly with views, followers, likes, comments, duration, bookmarks (strong), shares, and “coins”(strong)—all moderate except where noted (*p <* 0.05). GQS showed moderate links with followers and duration, and weak links with likes, comments, bookmarks, and coins (*p <* 0.05). mDISCERN was weakly associated only with follower count and duration (*p <* 0.05). Across both platforms, correlation coefficients clustered between 0.2 and 0.7, indicating consistently positive, low-to-moderate relationships (see Table 5).

**Table 5:** Spearman correlation analysis of video quality and audience interaction between YouTube and Bilibili.

Platform		YouTube (*N =* 80)			Bilibili (*N =* 77)		
Scores		VIQI	GQS	mDISCERN	VIQI	GQS	mDISCERN
Views	r	0.452	0.133	0.019	0.461	0.165	−0.114
	*p*	0	0.241	0.87	0	0.152	0.324
Fan	r	0.298	−0.011	−0.052	0.569	0.451	0.28
	*p*	0.007	0.92	0.645	0	0	0.014
Thumbs-up	r	0.515	0.223	0.059	0.587	0.246	−0.045
	*p*	0	0.047	0.601	0	0.031	0.7
Comments	r	0.399	0.103	−0.053	0.555	0.304	0.021
	*p*	0	0.366	0.639	0	0.007	0.857
Video length	r	0.556	0.424	0.436	0.45	0.452	0.228
	*p*	0	0	0	0	0	0.047
Collections	r	NA	NA	NA	0.604	0.307	−0.009
	*p*				0	0.007	0.937
Shares	r	NA	NA	NA	0.473	0.159	−0.140
	*p*				0	0.166	0.223
Coin	r	NA	NA	NA	0.607	0.311	0.037
					0	0.006	0.75

P, *P* < 0.05 were considered statistically significant. NA, Not Available.

## Discussion

Anxiety and depression among university students are global phenomena (14, 15), yet their drivers differ markedly across cultures. By using YouTube and Bilibili as lenses, we provide the first systematic comparison of how the two platforms frame—and sometimes distort—student mental health.

Our findings show that Bilibili consistently outperforms YouTube in views, likes, and comments. One reason is rooted in origin: Bilibili was built for young Chinese users, a cohort that remains highly active and prone to repeated, almost addictive, engagement (16). For many university students, part of the pleasure is interacting with the video itself—witty, meme-filled comment threads invite others to join the conversation. Eye-catching thumbnails further nudge viewers to click. Metrics unique to Bilibili—favourites, shares, and “coin” tips—cluster around verified creators with large followings, suggesting that prestige on the platform drives these extra forms of endorsement.

YouTube channels carry markedly larger follower bases than their Bilibili counterparts. On YouTube, the television-style or mini-documentary format (*N =* 41) attracts the most subscribers and is over-represented among verified accounts. Bilibili, in contrast, is dominated by short, low-budget clips uploaded by individual university students; these videos reach smaller audiences and are often less polished. Verification rates differ accordingly: 43.75% (35/80) of sampled YouTube videos came from certified accounts versus 28.57% (22/77) on Bilibili. Both platforms rely mainly on “personal” verification, yet licensed psychologists remain scarce—only five on YouTube and three on Bilibili. Expanding the pool of qualified mental-health professionals who post in either language would increase both the volume and credibility of videos addressing anxiety and depression among university students.

We classified each video by style. YouTube content chiefly follows a television or documentary format, whereas Bilibili favours single-speaker narratives (*N =* 25) and animated or motion-graphic clips (*N =* 23). English-language videos typically repackage broadcast segments; Chinese-language creators prefer self-recorded talks or bespoke animations to educate university students about mental health.

YouTube outscored Bilibili on VIQI, GQS, and mDISCERN because each platform’s DNA rewards different behaviours. YouTube’s “long-form + search + subscribe” triangle lets documentary-style, TV-derived content recoup high production costs through multi-slot advertising, so creators who invest in rigorous, evidence-based mental-health education are directly paid for quality (17). Bilibili’s bullet-comment ecosystem privileges speed and emotional punch; creators maximise income by triggering instant resonance, so storytelling and drama routinely outrank scientific precision (18). In short, YouTube aligns revenue with watch-time and authority, whereas Bilibili aligns it with real-time interaction—hence the systematic quality gap.

On Bilibili, likes, favourites, and “coins” rose in tandem with VIQI, yet absolute scores still trailed YouTube, showing that high engagement is not high quality. Students struggling with anxiety or depression often exhibit narrowed cognition and confirmation bias, making them more receptive to emotional appeal than to evidence (19). If algorithms continue to rank by interaction alone, the platform risks a feedback loop: the more distressed the viewer, the more they click on sensational content, and the further their misconceptions are reinforced. Previous work shows that misleading mental-health videos delay professional help and can deepen self-stigma (20). Platforms should therefore add a “quality-adjustment factor”that weights verified credentials, DISCERN scores, and factual accuracy, curbing preventable public-health harm.

Verified psychiatrists or clinical psychologists account for <6% of creators on either platform, far below the hospital-society-creator partnerships common in Europe and North America. Chinese university counselling centres house ample expertise, yet staff are judged by research output, not outreach, leaving the content arena to “emotional self-media” accounts whose credentials are often thin. We recommend that education authorities add “high-quality mental-health education” to universities’ social-service metrics and fund mixed teams of licensed clinicians, faculty, and students to run official channels. Co-creation with students cuts production costs and can supply reliable videos at scale.

Although YouTube offers higher overall quality, its English-language, Western-oriented examples translate poorly to Chinese students. Bilibili’s “monologue + long-form” format earns strong engagement because creators speak in the first person, film on campus, and invoke locally salient stressors—graduate-school admission, the CET-4/6 exams, autumn job fairs—so viewers feel “one of us” and “seen.” Future interventions should therefore pair global evidence with local narrative: keep the core content evidence-based (e.g., CBT skills, mindfulness steps) but wrap it in settings unique to Chinese universities—GPA curves, dormitory culture, parental expectations—to maximize acceptability and behavioural uptake.

This study has the following limitations: ① Platform asymmetry: YouTube does not provide indicators such as “donations,” “favorites,” and “reposts,” making it impossible to directly compare with Bilibili’s native interaction system; ② Insufficient granularity: Both platforms round or hide the numbers of views, likes, and followers, allowing only approximate comparisons, which may lead to measurement bias; ③ Missing classification: The disclosure rate of professional certifications (such as clinical psychologist licenses) is extremely low, limiting the stratified analysis of the authors’ true professional level; ④ Time and space limitations: The sample is a single cross-sectional data and is concentrated in the Chinese and English contexts; social media is evolving rapidly, and the conclusions may change within a few months; ⑤ Limited platform scope, only including YouTube and Bilibili; future research can be expanded to the international version of TikTok and include short video applications such as Xiaohongshu, Douyin, or Kuaishou in the Chinese context.

## Conclusion

A horizontal comparison of 157 videos from YouTube and Bilibili confirmed that the platform’s characteristics determine the quality of the content, rather than audience popularity. YouTube, with its long videos, institutionalized production, and advertising sharing mechanism, significantly outperformed Bilibili in all dimensions of VIQI, GQS, and mDISCERN. Although Bilibili won high user retention through bullet-screen interaction and local narratives among college students, it also diluted scientific rigor, forming a potential closed loop of “more anxiety→more emotional→more reinforcement of misinformation.” The proportion of practicing psychiatrists/psychologists on both platforms was less than 6%. The loss of professional voice is a common cross-cultural issue. Crucially, in the English-speaking context of YouTube,"documentaries-expert explanations” convey universal evidence-based solutions, while in the Chinese-speaking context of Bilibili, “campus monologues, and bullet-screen empathy” encapsulate local pressure issues. The two platforms are complementary rather than substituting in terms of cultural adaptation and professional depth. Therefore, high-quality mental health science popularization must introduce a “quality correction coefficient” to restructure algorithm weights and continuously output evidence-based content through the “official professional account + student co-creation” model, so as to transform high interaction into high effectiveness and prevent digital platforms from causing further harm to college students’ anxiety.

## Data Availability

The raw data supporting the conclusions of this article will be made available by the authors, without undue reservation.
